# Multiscale Modeling of Polycrystalline NiTi Shape Memory Alloy under Various Plastic Deformation Conditions by Coupling Microstructure Evolution and Macroscopic Mechanical Response

**DOI:** 10.3390/ma10101172

**Published:** 2017-10-13

**Authors:** Li Hu, Shuyong Jiang, Tao Zhou, Jian Tu, Laixin Shi, Qiang Chen, Mingbo Yang

**Affiliations:** 1 College of Material Science and Engineering, Chongqing University of Technology, Chongqing 400054, China; zt19811118@cqut.edu.cn (T.Z.); tujian@cqut.edu.cn (J.T.); shilaixin2016@cqut.edu.cn (L.S.); yangmingbo@cqut.edu.cn (M.Y.); 2 College of Mechanical and Electrical Engineering, Harbin Engineering University, Harbin 150001, China; jiangshy@sina.com; 3 Southwest Technology and Engineering Research Institute, Chongqing 400039, China; 2009chengqiang@163.com; 4 Precision Forming Integrated Manufacturing Technology of Collaborative Innovation Center, Chongqing 400039, China

**Keywords:** shape memory alloy, plastic deformation, crystal plasticity, finite element method, multiscale modeling

## Abstract

Numerical modeling of microstructure evolution in various regions during uniaxial compression and canning compression of NiTi shape memory alloy (SMA) are studied through combined macroscopic and microscopic finite element simulation in order to investigate plastic deformation of NiTi SMA at 400 °C. In this approach, the macroscale material behavior is modeled with a relatively coarse finite element mesh, and then the corresponding deformation history in some selected regions in this mesh is extracted by the sub-model technique of finite element code ABAQUS and subsequently used as boundary conditions for the microscale simulation by means of crystal plasticity finite element method (CPFEM). Simulation results show that NiTi SMA exhibits an inhomogeneous plastic deformation at the microscale. Moreover, regions that suffered canning compression sustain more homogeneous plastic deformation by comparison with the corresponding regions subjected to uniaxial compression. The mitigation of inhomogeneous plastic deformation contributes to reducing the statistically stored dislocation (SSD) density in polycrystalline aggregation and also to reducing the difference of stress level in various regions of deformed NiTi SMA sample, and therefore sustaining large plastic deformation in the canning compression process.

## 1. Introduction

Owing to its functional properties, including shape memory effect, superelasticity, and biological compatibility, NiTi shape memory alloy (SMA) has attracted increasing attention in the field of materials science and engineering. It is generally accepted that hot plastic deformation, rather than cold plastic deformation, plays a significant role in transforming as-cast NiTi SMA ingots into NiTi SMA products, including NiTi wire, bar, tube, strip, and sheet [1,2,3]. On the one hand, hot plastic deformation of NiTi SMA has advantages in obtaining the perfect microstructure during manufacturing the products of NiTi SMA, which in turn has a significant influence on the mechanical and functional properties of NiTi SMA [4,5]. On the other hand, cold plastic deformation of NiTi SMA has an adverse influence on manufacturing NiTi SMA workpieces since NiTi SMA possesses very poor plasticity at room temperature [6,7,8]. Therefore, for the purpose of further broadening the engineering application of NiTi SMA, it is necessary to perform a deep insight into deformation mechanisms and microstructure evolution during the process of hot plastic deformation of NiTi SMA. Various experimental investigations in terms of deformation mechanisms and microstructure evolution of NiTi SMA have been conducted, and the corresponding results have confirmed that plastic deformation mechanisms of NiTi SMA are temperature-dependent and there exhibits a predominant distinction in the case of various temperatures, including stress-induced martensite phase transformation, dislocation slip, deformation twinning, grain boundary slide, grain rotation, dislocation climb, and grain boundary migration [9,10]. However, so far, many theoretical analyses and numerical simulations have focused on coupling plasticity and phase transformation of NiTi SMA [11,12,13]. In terms of simulating hot plastic deformation of NiTi SMA, our literature search has shown that both the microscale and macroscale simulations have not been fully addressed yet.

Historically, the finite element method (FEM), as a numerical method, is the main approach applied in the industry to simulate large scale forming processes. This method is a perfect candidate for clarifying the plastic deformation law of a metallic alloy by describing material behavior as a continuum [14,15]. However, as the major assumption of FEM is that the behavior and interaction of individual grains in large scale problems are homogenized in the form of a single flow stress model, FEM, which is based on macroscopic constitutive theory, is unable to capture the microstructure evolution and texture evolution of polycrystalline metallic alloy subjected to plastic deformation. The aforementioned problem has been successfully overcome by incorporating the crystal plasticity theory with FEM, forming the crystal plasticity finite element method (CPFEM). Via using CPFEM, various studies have been conducted in terms of revealing plastic deformation mechanisms and inhomogeneous deformation of metallic alloy during plastic deformation [16,17,18,19,20]. Specifically, during these numerical studies, the mainly adopted boundary conditions are either periodic boundary conditions (PBCs) or just keep all surface plane. It is worth mentioning that these two kinds of boundary conditions are both the simplification of interactions between the constructed polycrystalline model and neighboring grains. Therefore, in order to more realistically reflecting the microstructure evolution during plastic deformation of polycrystalline metallic alloy, boundary conditions that can effectively link the microscale and macroscale are needed, particularly in the case of complex deformation process. 

In the present study, numerical modeling of microstructure evolution in various regions during uniaxial compression and canning compression of NiTi SMA are conducted through combined macroscopic and microscopic finite element simulation in order to investigate plastic deformation of NiTi SMA at 400 °C. The corresponding deformation history in some selected regions of the macroscopic finite element model is directly extracted by means of the sub-model technique of finite element code ABAQUS and subsequently used as the boundary conditions for the microscale simulation by means of CPFEM.

## 2. Modeling Methodology

**Figure 1:**
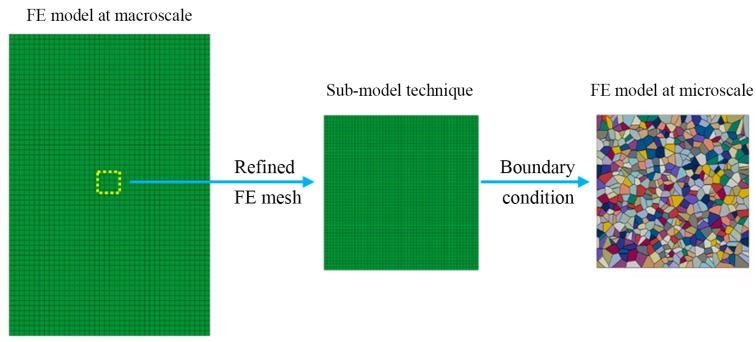
Schematic diagram of the idea of the multiscale method based on the sub-model technique of finite element code ABAQUS.

Figure 1 shows a schematic illustration of the multiscale method used in the present study. In this approach, the macroscale finite element model is used as a global model with relatively coarse finite element mesh and the overall response of the polycrystalline material is obtained by the finite element homogenization, as well as the strain distribution and stress distribution. Afterward, some regions of interest are then selected in this finite element mesh, and the corresponding deformation history in these selected regions of finite element model is directly extracted by means of the sub-model technique of finite element code ABAQUS. Further details about sub-model technology can be found in [21]. Finally, using this extracted deformation history as boundary conditions, numerical modeling of microstructure evolution at the microscale level is conducted with a refined finite element mesh.

### 2.1. Macroscale Finite Element Model

**Figure 2:**
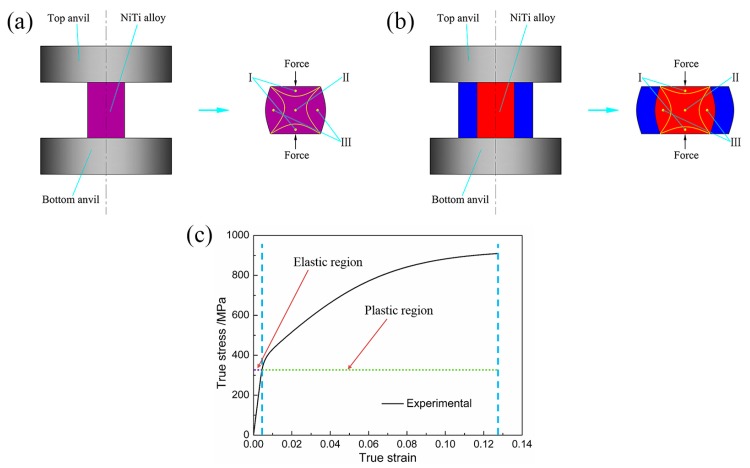
Schematic diagram of NiTi SMA under: (**a**,**b**) uniaxial compression process and canning compression process, where zone I showing the minimum deformation zone, zone II showing the principal deformation zone and zone III showing the intermediate deformation zone; (**c**) material response as the input data of macroscale finite element simulation based on experimental result of NiTi SMA subjected to uniaxial compression at 400 °C at the constant strain rate of 0.001 s^−1^.

Figure 2 shows the schematic diagrams of uniaxial compression and canning compression of NiTi SMA samples with the diameter of 4 mm and the height of 6 mm. Moreover, the NiTi SMA samples were prepared from the as-received NiTi SMA bar with a nominal composition of Ni_50.9_Ti_49.1_ (at.%) by means of electro-discharge machining (EDM). In addition, the as-received NiTi SMA bar was prepared by virtue of vacuum induction melting method and subsequent rolling at 800 °C. In the case of canning compression, the NiTi SMA sample was canned in the low carbon steel cans with the inner diameter of 4 mm and the outer diameter of 10 mm. The elastic and plastic data used in macroscale finite element simulation is directly obtained from the mechanical response of NiTi SMA subjected to uniaxial compression at 400 °C at the constant strain rate of 0.001 s^−1^ by using an INSTRON-5500R universal testing machine (Instron Corporation, Norwood, MA, USA) equipped with a heating device. In this way, this model provides general distribution of displacement, strain and stress at the macroscale level.

### 2.2. Microscale Finite Element Model

**Figure 3:**
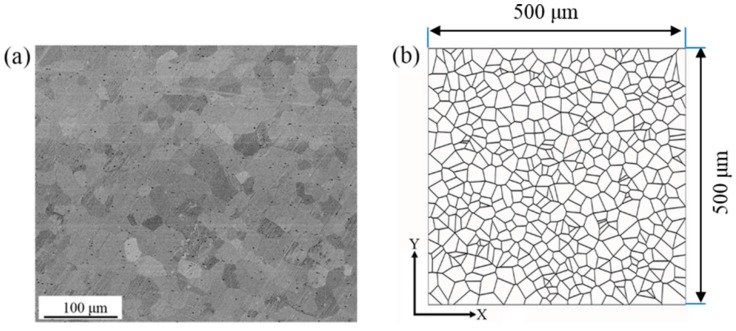
(**a**) Cross-sectional scanning electron microscope morphology of as-received NiTi SMA and (**b**) voronoi tessellation generated using NEPER.

In the present study, interest is mainly focus on understanding the heterogeneous deformation and microstructure evolution in the selected regions with the corresponding boundary conditions extracted from the macroscale finite element simulation. Therefore, a polycrystalline model with refined mesh is constructed and used, since the accurate representation of grain shape and especially grain boundary is important in capturing the heterogeneous deformation in polycrystal. To acquire the initial microstructure and texture of NiTi SMA, a scanning electron microscope (SEM) experiment and an electron back-scattered diffraction (EBSD) experiment were conducted on the as-received specimen using a Zeiss Supra 55 scanning electron microscope (SEM, Carl Zeiss Company, Oberkochen, Germany) coupled with OXFORD EBSD instrument (Oxford Instruments, Oxford, UK). By applying the intercept method on individual grains, it has been confirmed that the as-received NiTi SMA are composed of equiaxed grains with an equivalent grain diameter of about 25 μm. Based on this experimental result, the polycrystalline model on the basis of Voronoi tessellation is constructed by means of a free open source software package NEPER [22], as shown in Figure 3, where the microscale model contains 512 grains and its model size is 500 μm × 500 μm. Moreover, the corresponding microscale model constructed in finite element code ABAQUS contains 130915 plane strain elements. This refined mesh contributes to improving the simulation accuracy at the microscale.

### 2.3. Crystal Plasticity Constitutive Model

In the present study, the framework of crystal plasticity theory is based on dislocation slip, and three slip systems, including {110}<100>, {010}<100>, and {110}<111>, are introduced into the proposed crystal plasticity finite element model [9]. As it has been confirmed that dislocation slip, rather than deformation twinning, is responsible for the plastic deformation of NiTi SMA subjected to compression deformation at 400 °C [10]. Moreover, it can be generally accepted that 400 °C is higher than the martensite desist temperature, above which stress-induced martensite phase transformation does not take place [9].

In the framework of rate-dependent single crystal plasticity, the elastic constitutive equation is specified by the following equation [23,24].

(1) $$\overset{\nabla *}{\sigma }\;+\sigma \;(I:D^{*})=\;L:D^{*}$$

where I is the second order identity tensor, L is the tensor of elastic modulus having the full set of symmetries $L_{ijkl}=L_{jikl}=L_{ijlk}=L_{klij}$, $D^{*}$ is the symmetric stretching rate of the lattice. The Jaumann rate $\overset{\nabla *}{\sigma }$ is the corotational stress rate on the axes that rotate with the crystal lattice, and it is related to the corotational stress rate on the axes that rotate with the material $\overset{\nabla }{\sigma }$ by the following equation.

(2) $$\overset{\nabla *}{\sigma }\;=\overset{\nabla }{\sigma }+\Omega^{p}\cdot \sigma -\sigma \cdot \Omega^{p}$$

where $\overset{\nabla }{\sigma }=\dot{\sigma }-\Omega \cdot \sigma +\sigma \cdot \Omega$. In addition, Ω and $\Omega^{p}$ are the total lattice spin tensor and plastic part of the total lattice spin tensor, respectively. 

The crystal was assumed to behave as an elasto-viscoplastic solid, so the slipping shear rate ${\dot{\gamma }}^{\alpha }$ in individual α slip system is of great importance in crystal plasticity calculation. Based on the Schmid law, the slipping shear rate ${\dot{\gamma }}^{\alpha }$ can be determined by a simple rate-dependent power law relation, namely

(3) $${\dot{\gamma }}^{\alpha }={\dot{\gamma }}_{0}{\left|\tau^{\alpha }/g^{\alpha }\right|}^{n}\;sign(\tau^{\alpha }/g^{\alpha })$$

where *n* stands for the rate dependency. If the material is rate-independent, a large value can be chosen up to 50, whereas if the material is highly rate-dependent, a typical value of 10 can be used [19]. In the present study, the value of n is chosen to be 20, indicating a certain rate dependency as reported in [25]. ${\dot{\gamma }}_{0}$ is a reference shear strain rate. $\tau^{\alpha }$ and $g^{\alpha }$ are resolved shear stress on the slip system α and slip resistance of this system, respectively. Furthermore, the change rate of slip resistance in each slip system is given as follows:

(4) $${\dot{g}}^{\alpha }=\sum_{\beta }^{n}h_{\alpha \beta }{\dot{\gamma }}^{\beta }$$

where $h_{\alpha \beta }$ are the slip hardening modulus, and the sum operation is performed over all the activated slip systems. Here, $h_{\alpha \alpha }$ is known as self-hardening modulus and it is derived from the hardening of slip system itself. In addition, $h_{\alpha \beta }$ (α≠β) is called latent-hardening modulus, indicating that the hardening is caused by another slip system. 

A simple hardening model is given as follows [26]:

(5) $$\begin{array}{l} h_{\alpha \alpha }=h(\gamma )=h_{0}\sec h^{2}\left|\frac{h_{0}\gamma }{\tau_{s}-\tau_{0}}\right|,\;\gamma =\sum_{\alpha }\int_{0}^{t}\left|{\dot{\gamma }}^{\alpha }\right|dt\\ h_{\alpha \beta }=qh(\gamma )\;(\alpha \neq \beta ) \end{array}$$

where $h_{0}$ is the initial hardening modulus, $\tau_{0}$ is the initial yield stress, $\tau_{s}$ is the saturation stress, γ is the total shear strain in all the slip systems, and q is the ratio of latent-hardening to self-hardening and *q* = 1.4 is used in the present study [18].

The aforementioned crystallographic formulations are implemented numerically into ABAQUS standard solver through a user-defined material subroutine (UMAT), where the implicit (Euler backward) integration algorithm is adopted [24]. Moreover, the initial orientations of individual grains are obtained by discretizing the orientation distribution function (ODF) of EBSD data. Further details of orientation discretization and assignment can be found in [27]. The set of constitutive parameters used in the present study is shown in Table 1, where the elastic constants of NiTi SMA are obtained from the literature [9]. These remaining parameters are identified by a “trial-error” procedure in back-fitting the mechanical result in the polycrystalline model to the mechanical response in uniaxial compression experiment. In general, a “trial-error” procedure is time consuming, but its results with respect to parameter identification are not as “improper” as the term might indicate. 

## 3. Results and Discussion

### 3.1. Distribution of Stress and Strain at Macroscale

**Figure 4:**
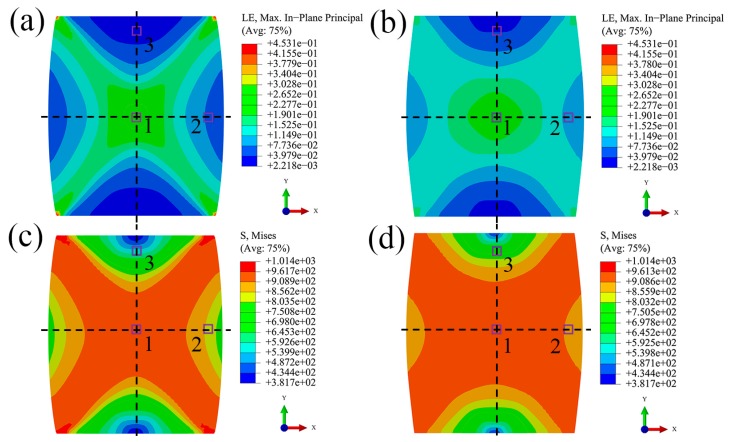
Macroscale strain and stress distribution of NiTi SMA at the deformation degree of 12%: (**a**,**c**) under uniaxial compression process; (**b**,**d**) under canning compression process. (Regions 1, 2, and 3 of interest correspond to three characteristic deformation zones during plastic deformation.)

Figure 4 shows the macroscale simulation results of uniaxial compression and canning compression of NiTi SMA at the deformation degree of 12%. It is obvious that distribution of stress and strain is inhomogeneous at the macroscale in these two deformation processes. Moreover, this inhomogeneity in the case of canning compression is relatively weaker than in the case of uniaxial compression. This phenomenon is originated from the fact that in the case of canning compression, the compression deformation of the low carbon steel can lead to the increase of the compressive loading on the surfaces of the NiTi SMA sample, then results in a three-dimensional compressive loading state of the deformed NiTi SMA sample, which finally relieves the inhomogeneity of plastic deformation during canning compression. In order to investigate the microstructure evolution of NiTi SMA in various boundary conditions and in various deformation processes, three characteristic regions shown in Figure 4 are selected, where region 1 corresponds to the region with the maximal plastic deformation, region 2 corresponds to the region with the in-between plastic deformation, and region 3 corresponds to the region with the minimal plastic deformation. The corresponding deformation history is extracted by means of the sub-model technique of finite element code ABAQUS and then applied at the microscale simulation by means of CPFEM.

### 3.2. Distribution of Stress and Strain at Microscale

**Figure 5:**
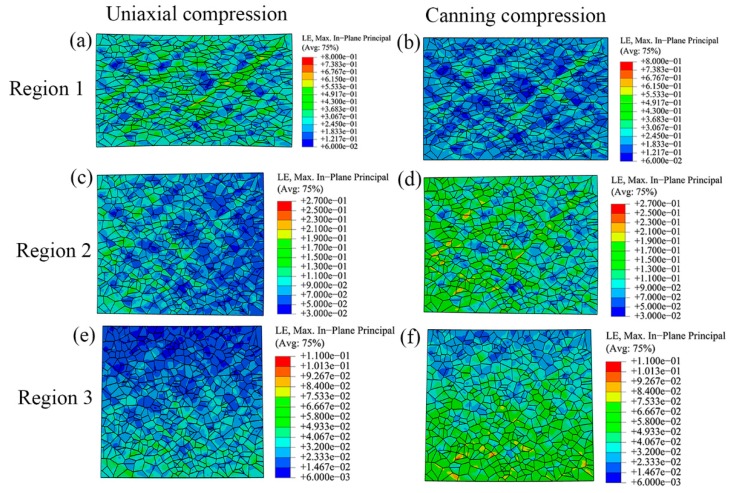
Strain distribution at the microscale level during uniaxial compression and canning compression processes with the locations of the polycrystalline models corresponding to regions 1, 2, and 3 in Figure 4.

**Figure 6:**
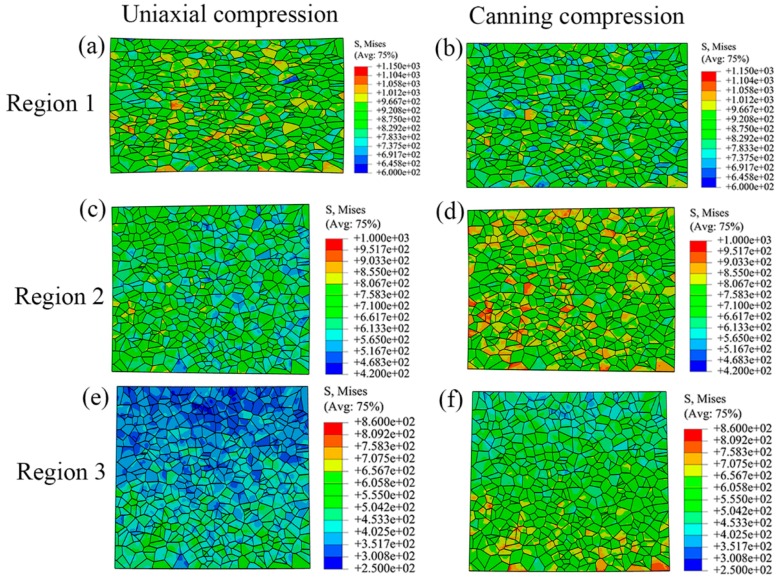
Stress distribution at the microscale level during uniaxial compression and canning compression processes with the locations of the polycrystalline models corresponding to regions 1, 2, and 3 in Figure 4.

Figure 5 shows the strain distribution of polycrystalline model subjected to various boundary conditions during uniaxial compression and canning compression. Inhomogeneity at the microscale is also observed in these two deformation processes. In region 1, shear bands in the polycrystalline model in the case of uniaxial compression are with larger plastic strain than in the case of canning compression. Moreover, the polycrystalline model in the case of uniaxial compression undergoes larger elongation along radial direction than in the case of canning compression. In regions 2 and 3, the polycrystalline models in the case of canning compression have larger plastic strain than in the case of uniaxial compression. These observations are due to the effect of the low carbon steel can in the case of canning compression, which provides compressive load in the radial direction of NiTi SMA sample and therefore contributes to relieving the inhomogeneous plastic deformation in various regions and enhancing the plastic deformation in regions 2 and 3. As a result, canning compression possesses less inhomogeneity than uniaxial compression. Similar to the strain distribution, the stress distribution of the polycrystalline model subjected to various boundary conditions also shows high inhomogeneity, as shown in Figure 6. In region 1, as the strain inhomogeneity is relieved in the case of canning compression, the stress distribution is more homogeneous than the corresponding stress distribution in the case of uniaxial compression. However, in regions 2 and 3, as polycrystalline models sustain more plastic deformation in the case of canning compression than in the case of uniaxial compression, the stress level in the case of canning compression is higher than the corresponding stress level in the case of uniaxial compression, which results in reducing the difference of stress level in various regions. Moreover, stress concentration near the grain boundaries has been more frequently observed in the case of canning compression than in the case of uniaxial compression. It should be noted that in both deformation processes, the maximum value in legends corresponding to regions 2 and 3 are lower than the maximum value in legends corresponding to region 1. This issue is due to the fact that the polycrystalline models corresponding to regions 2 and 3 sustain less plastic strain than the one corresponding to region 1 during plastic deformation.

### 3.3. Distribution of Dislocation at Microscale

On the one hand, dislocation is the vehicle to sustain plastic deformation. On the other hand, region 1 possesses the maximal plastic strain. Therefore, polycrystalline models corresponding to region 1 have the highest dislocation density during uniaxial compression and canning compression. In order to investigate the distribution of statistically stored dislocation (SSD) density during uniaxial compression and canning compression of NiTi SMA, the methodology described in [28] is adopted and SSD density can be calculated in each integration points according to the following equation.

(6) $$\rho_{SSD}={\left(\frac{2\bar{\tau }}{Gb}\right)}^{2}$$

where G is the shear module of NiTi SMA and b is the corresponding magnitude of the Burgers vector. $\bar{\tau }$ stands for the averaged resolved shear stress and it can be expressed by the following equation.

(7) $$\bar{\tau }=\frac{1}{N}\sum_{\alpha =1}^{N}\tau^{\alpha }$$

**Figure 7:**
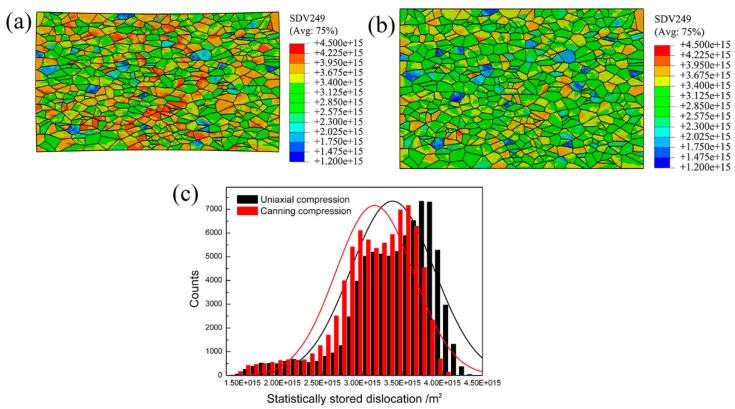
Contour plots of SSD density at the microscale level with the locations of the polycrystalline models corresponding to region 1 in Figure 4 during: (**a**) uniaxial compression process and (**b**) canning compression process; (**c**) Statistical analysis of SSD density in (**a**,**b**).

Figure 7 shows the SSD density distribution of polycrystalline models subjected to various boundary conditions in region 1 in the case of uniaxial compression and canning compression. These simulated results indicate that the spatial distribution of SSD density in region 1 is highly inhomogeneous in there two deformation processes. It can be noted that in the case of uniaxial compression, individual grains have higher SSD density than in the case of canning compression. This issue can be further confirmed by the statistical analysis of SSD density, as shown in Figure 7c where the average SSD density is 3.38 × 10^15^ m^−2^ in the case of uniaxial compression and the corresponding value in the case of canning compression is 3.15 × 10^15^ m^−2^. This can be served as an evidence that at the same deformation degree, canning compression contributes to relieving the SSD density within the deformed sample than uniaxial compression. As a result, canning compression is favorable of sustaining large plastic deformation.

## 4. Conclusions

Due to the aforementioned analysis, the results obtained from the macroscale numerical modeling by FEM and the microscale numerical modeling on the basis of CPFEM contribute to presenting a clear understanding of microstructure evolution in various regions during uniaxial compression and canning compression.

(1) At the macroscale, it can be concluded that canning compression contributes to relieving the inhomogeneous plastic strain as the compression of the low carbon steel can results in a three-dimensional compressive loading state of NiTi SMA sample.
(2) The sub-model technique in the finite element code ABAQUS is used to extracted the accurate deformation history of selected regions at the macroscale simulation. Then, this deformation history, which reflects the complex interaction between the polycrystalline aggregation and neighboring grains, can serve as boundary conditions used at the microsacle simulation.
(3) At the microscale, it can be concluded that the mitigation of inhomogeneous plastic deformation in canning compression contributes to various microstructure evolution in selected regions. Moreover, the ease of inhomogeneity in plastic strain contributes to reducing the difference of the stress level in various regions and reducing statistically stored dislocation (SSD) density within the deformed NiTi SMA sample. Therefore, canning compression is favorable of sustaining large plastic deformation.

## Figures and Tables

**Table 1:** Material parameters of as-received NiTi SMA.

$C_{11}$	$C_{12}$	$C_{44}$	$h_{0}$	$\tau_{s}$	$\tau_{0}$	${\dot{\gamma }}_{0}$	*q*	*n*
130 GPa	98 GPa	34 GPa	1200 MPa	322 MPa	160 MPa	0.001 s^−1^	1.4	20
